# Adipose tissue‐derived extracellular matrix hydrogels as a release platform for secreted paracrine factors

**DOI:** 10.1002/term.2843

**Published:** 2019-04-15

**Authors:** Joris A. van Dongen, Vasilena Getova, Linda A. Brouwer, Gabriel R. Liguori, Prashant K. Sharma, Hieronymus P. Stevens, Berend van der Lei, Martin C. Harmsen

**Affiliations:** ^1^ Department of Pathology and Medical Biology University of Groningen, University Medical Center Groningen Groningen The Netherlands; ^2^ Department of Plastic Surgery University of Groningen, University Medical Center Groningen Groningen The Netherlands; ^3^ Laboratory of Cardiovascular Surgery and Circulation Pathophysiology (LIM‐11) Heart Institute (InCor), Hospital das Clinicas, Faculdade de Medicina, Universidade de Sao Paulo Sao Paulo Brazil; ^4^ Department of Biomedical Engineering University of Groningen, University Medical Center Groningen Groningen The Netherlands; ^5^ Bergman Clinics Den Haag The Netherlands; ^6^ Bergman Clinics Heerenveen, Zwolle, and Groningen The Netherlands

**Keywords:** acellular matrix, decellularization, growth factors, hydrogel, mesenchymal stem cells, wound healing

## Abstract

Fat grafting is an established clinical intervention to promote tissue repair. The role of the fat's extracellular matrix (ECM) in regeneration is largely neglected. We investigated in vitro the use of human adipose tissue‐derived ECM hydrogels as release platform for factors secreted by adipose‐derived stromal cells (ASCs). Lipoaspirates from nondiabetic and diabetic donors were decellularized. Finely powdered acellular ECM was evaluated for cell remainders and DNA content. Acellular ECM was digested, and hydrogels were formed at 37°C and their viscoelastic relaxation properties investigated. Release of ASC‐released factors from hydrogels was immune assessed, and bio‐activity was determined by fibroblast proliferation and migration and endothelial angiogenesis. Acellular ECM contained no detectable cell remainders and negligible DNA contents. Viscoelastic relaxation measurements yielded no data for diabetic‐derived hydrogels due to gel instability. Hydrogels released several ASC‐released factors concurrently in a sustained fashion. Functionally, released factors stimulated fibroblast proliferation and migration as well as angiogenesis. No difference between nondiabetic and diabetic hydrogels in release of factors was measured. Adipose ECM hydrogels incubated with released factors by ASC are a promising new therapeutic modality to promote several important wound healing‐related processes by releasing factors in a controlled way.

## 1 INTRODUCTION

Autologous fat grafting has been widely investigated and used for soft‐tissue defects and regenerative purposes such as the treatment of open wounds as well as antiscarring treatments (Han, Kim, & Kim, 2010; Jaspers et al., 2017; Negenborn, Groen, Smit, Niessen, & Mullender, 2016; Stasch et al., 2015). Diabetes is associated with dysfunctional wound healing and occurrence of nonhealing ulcers. A significant proportion of diabetics does not respond to revascularization therapies, which regularly results in amputation of the affected limb. Few noncontrolled clinical trials and case reports have shown that diabetic wound healing is augmented by lipografting (Cervelli et al., 2010; Han et al., 2010; Stasch et al., 2015; Vicenti et al., 2017). One of the key components of this regenerative potential of adipose tissue that dictates tissue regeneration is the stromal vascular fraction (SVF), which is rich in adipose tissue‐derived stromal cells (ASCs). In the SVF, ASCs are present as pericytes or periadventital vascular cells (Corselli et al., 2012; Lin et al., 2008). The clinical efficacy of SVF is often ascribed to these resident ASC or other precursor cells, but definitive proof still lacks. Another important but neglected contributor to regeneration is the extracellular matrix (ECM), which provides the tissue architecture and structural support as well as key biochemical signalling cues. The role of ECM in tissue regeneration is but poorly studied, if at all. ASCs secrete a plethora of growth factors, cytokines, lipid‐based mediators, and matrix molecules such as structural components and matrix metalloproteases (MMPs) that remodel ECM. For instance, MMPs can degrade the excessively deposited ECM by myofibroblasts in scars, whereas vascular endothelial growth factors (VEGFs) and fibroblast growth factors (FGFs) promote wound healing (Spiekman et al., 2014; Spiekman et al., 2017). The ECM in SVF supports the proliferation and survival as well as differentiation of cells by binding of their surface‐expressed integrins to ECM molecules. Moreover, paracrine factors bind and are retained by proteoglycans and glycoaminoglycans of ECM. In this way, ECM stores paracrine factors and functions as a physiological controlled “on‐demand” release system (Mescher, 2010; van Dongen, Stevens, Harmsen & van der Lei, 2017).

Concentration of the SVF of adipose tissue, that is, to get rid it of the large amounts of adipocytes and thereby reducing volume, might augment the clinical observed regenerative potential compared with standard autologous fat grafting. To concentrate SVF, the SVF can be isolated enzymatically or mechanically (van Dongen, Tuin, et al., 2017). Enzymatic isolation destructs the ECM and its interaction with cells and thus yields a suspension of single SVF cells (cellular SVF or cSVF). Via centrifugation, adipocytes are readily removed with enzymatic isolation (van Dongen, Tuin, et al., 2017). When a nonenzymatic isolation procedure is used, adipocytes are mechanically destructed and the ECM is still intact as are its interactions with bound cells, and it also acts for sustained release of bound factors (tissue‐like SVF or tSVF) (van Dongen, Stevens et al., 2017; van Dongen, Tuin, et al., 2017). Therefore, we anticipate a nonenzymatically obtained SVF to have a higher therapeutic benefit than enzymatic SVF or unprocessed lipografts.

Systemic, often chronic, diseases such as diabetes mellitus (DM) impact the clinical efficacy of autologous SVF injections. For DM, long‐term hyperglycaemic exposure of ASC and ECM influences their biological properties (Haucke, Navarrete‐Santos, Simm, Silber, & Hofmann, 2014; Serban et al., 2015; Voziyan, Brown, Chetyrkin, & Hudson, 2014). High levels of glucose impair the regenerative function of ASC by intracellular reactive oxygen species accumulation (Peng et al., 2017). Reactive oxygen species inhibits the proliferation and proangiogenic capacities of ASC, which are important processes in wound healing (Peng et al., 2017). Furthermore, high levels of glucose and their glycolytic products such as methylglyoxal result in glycation of proteins. These so‐called advanced glycation end products (AGEs) bind to a receptor of AGE (RAGE) that is expressed by virtually all tissue cells and causes proinflammatory activation. Inflammation is relevant for wound repair, but increased chronic inflammation acts adverse and results in decreased vascularization and tissue necrosis as observed in diabetic ulcers. Glycation affects ECM molecules rather than circulating proteins due to their slow turnover, that is, long half‐life (Verzijl et al., 2000). Elevated AGEs damage ECM potentially in three different critical sites: compromised cellular binding site in the ECM, which inhibits cell adhesion, survival, and proliferation. Second, molecular sites in the ECM that are involved in turnover (both proteolytic degradation and cross linking) may be affected, which may result in adverse ECM remodelling, accumulation, changes in mechanical features, and associated changes of biological responses of bound tissue cells (Mott et al., 1997; Pozzi et al., 2009; Voziyan et al., 2014). Affected subunits of ECM molecules lead to more cross links between proteins and thus an unwanted higher stability and stiffness of ECM proteins (Voziyan et al., 2014). In soft tissues, increased stiffness promotes fibrotic processes in a feed‐forward looped fashion.

For those reasons, nondiabetic SVF is likely to be more suitable than diabetic SVF as treatment for, for example, wound healing of diabetic ulcers in diabetic patients. This would, however, implicate an allogeneic tissue transplantation, which will result in an immunological rejection of the administrated SVF. We surmised that the SVF's biological activity is primarily contained within the ECM and its bound paracrine factors in which parenchymal and stromal cells no more than serve to continuously replenish this biological entity. In particular, for short(er) term applications such as wound healing, ECM loaded with paracrine factors might show therapeutic efficacy. ECM can be obtained by decellularization of (adipose) tissue. Literature is littered with a host of decellullarization protocols that often dictate the use of detergents such as sodium dodecyl sulphate (SDS) as well as acids and bases to remove cellular constituents. This procedure not only removes all cell types but also the paracrine factors (Crapo, Gilbert, & Badylak, 2011). A decellularized ECM that is devoid of cells and paracrine factors is of reduced clinical value compared with mechanically derived SVF in which both ECM and paracrine factors are retained. Therefore, recharging of ECM with factors, for example, released from cultured ASC might restore a comparable clinical effect as mechanically derived SVF while preserving the option for interpatient administration.

In this study, we interrogated uptake and release of ASC‐released paracrine factors by human adipose tissue‐derived ECM hydrogels in vitro. The prepared adipose ECM hydrogels were first incubated with factors released by ASC, that is, conditioned culture medium and subsequently studied for wound healing purposes. Additionally, structural differences between diabetic and nondiabetic ECM regarding binding of factors were investigated.

## 2 MATERIAL AND METHODS

### 2.1 Processing and decellularization of adipose tissue

Adipose tissue was harvested from the abdomen of nondiabetic (*n* = 5) and diabetic (*n* = 5) patients during regular liposuction procedures and processed anonymously using the FAT procedure, as previously described by van Dongen, Stevens, Parvizi, van der Lei, and Harmsen (2016). Informed consent was obtained according to the local ethical committee of the University Medical Center of Groningen. Briefly, 50‐ml tubes containing lipoaspirate were centrifuged at 960 *× g* at room temperature (RT) for 2.5 min. Next, 10 ml of centrifuged adipose tissue was pushed forward and back 30 times through a Luer‐to‐Luer connector with three holes of 1.4 mm. The fragmented adipose tissue was again centrifuged at 960 *× g* at RT for 2.5 min. This procedure resulted in 1 ml of tSVF, which was then decellularized, as previously described by Roehm, Hornberger, and Madihally (2016). Briefly, 10 ml of tSVF was mixed with 30 ml of a 50% ethanol/water mixture and frozen at −80°C and thawed at RT in 30 min for four cycles. Then tSVF was incubated in 0.05% trypsin/0.05‐mM ethylenediaminetetraacetic acid (EDTA; 1:1 *v*/*v*) for 1.5 hr at 37°C. Afterwards, samples were sonicated (70 W) in 0.5% SDS at 46°C for 20 min. Samples were then lyophilized and immersed in xylene (1:10 *w*/*v*) for 17 min. Next, samples were washed with 96% ethanol and incubated in DNAse solution (LS002007, Worthington; containing a final concentration of 30 μg/ml of DNAse in 1.3 mM of MgSO_4_ and 2 mM of CaCl_2_) for 24 hr. Finally, samples (acellular matrix) were lyophilized again and homogenized with the use of an UltraTurrax fragmer (PM Tamson Instruments) and stored at −80°C before used in experiments.

### 2.2 Histological characterization of acellular adipose matrix

One‐time centrifuged adipose tissue and tSVF (controls) as well as acellular matrix of nondiabetic (NAM) and diabetic patients (DAM; *n* = 3) were formalin fixed and embedded in paraffin. Four‐micrometre thickness sections were cut, deparaffinized, and then incubated overnight with 0.1 M of Tris/HCL buffer (pH 9.0) at 80°C and stained with antibody against Perilipin A (1:200, Abcam) to visualize adipocytes as previously described (van Dongen, Stevens, Parvizi, van der Lei, & Harmsen, 2016). Samples were visualized under a light microscope (Leica Microsystems, DM IL). A Masson's trichrome staining and haematoxylin and eosin (H&E) staining were performed on deparaffinized 4 μm of slides. Then samples were mounted and visualized under a light microscope (Leica Microsystems, DM IL).

### 2.3 DNA content measurement

NAM and DAM samples (*n* = 3) were used to assess presence of DNA. A solution containing 5 μl of proteinase K (2 U/mg; Sigma Aldrich, 3115828001), 50 μl of 10% SDS, and 500 μl of SE buffer (75 mM of NaCl, 25 mM of EDTA, and pH 8.0) was added to 10 mg of acellular matrix. Solutions were mixed gently and incubated overnight at 55°C. Then 6 M of NaCl and chloroform was added, and samples were thoroughly mixed with the use of a top‐over‐top rotator for 30 min. Samples were centrifuged at 650 *× g* at 20°C for 10 min. Next, supernatant was taken and mixed with ice‐cold isopropanol. Samples were centrifuged at 18,000 *× g* at 4°C for 5 min. Then supernatant was discarded, and pellet was washed with 70% ethanol. Afterwards, pellet was dissolved in TE buffer (containing 10 mM of Tris, 0.1 mM of EDTA, and pH 8.0). DNA was quantified with the use of a Nanodrop metre. Additionally, a DAPI/phosphate‐buffered saline (PBS) staining was used to stain nuclei. Samples were visualized under a fluorescence microscope (Leica Microsystems, DM IL).

### 2.4 Sulphated glycosaminoglycans measurement

The concentration of sulphated glycosaminoglycans (sGAG) was measured with the use of a 1,9‐dimethylmethylene blue assay according to the protocol of Farndale, Sayers, and Barrett (Farndale, Sayers, & Barrett, 1982). NAM and DAM samples (*n* = 3) were digested in 1% proteinase K/SE solution at 55°C for 24 hr. As a control, a standard solution of 10 μg/ml of chondroitin sulphate C (#C4384‐250 mg, Sigma‐Aldrich, St. Louis, MO) was used. After the addition of the 1,9‐dimethylmethylene blue staining solution, extinction was measured at 525 and 595 nm. Additionally, an Alcian blue staining was used to visualize the total amount of glycosaminoglycans in nondiabetic and diabetic adipose tissue as well as tSVF and NAM and DAM samples. Samples were visualized under a light microscope (Leica Microsystems, DM IL).

### 2.5 Cell isolation, characterization, and collection of conditioned medium of ASCs

Adipose tissue was harvested by normal liposuction procedures from healthy donors (*n* = 3) used for cell isolation. Briefly, samples were washed with PBS three times. Then 0.1% collagenase A/1% bovine serum albumin in PBS was added as dissociation medium. The samples with 0.1% collagenase A/bovine serum albumin in PBS were then stirred in a water bath at 37°C for 1.5 hr. Next, cells were placed in lysis buffer on ice for 5 min to disrupt all erythrocytes. Cells were then centrifuged and cultured at 37°C at 5% CO_2_ in humidified incubator in Dulbecco's Modified Eagle's Medium (DMEM; BioWhittaker Walkersville, MD: 10% fetal bovine serum [FBS], 1% L‐glutamine [L‐Glut], and 1% penicillin/streptomycin [P/S]). Medium was refreshed twice a week. After culture, cells were characterized for ASC characteristics. Cells were characterized based on CD marker surface expression using flow cytometry (CD29, CD31, CD44, CD45, CD90, and CD105), adipogenic, osteogenic, and smooth muscle cell differentiation capacity as well as colony formation capacity. Pooled nondiabetic ASCs (*n* = 3) of Passages 4–6 were used to prepare ASC conditioned medium (ASC‐CMe; containing DMEM, 1% L‐Glut, and 1% P/S or RPMI 1% L‐Glut, and 1% P/S). Medium was collected and filtered through a 0.22‐μm filter after 24 hr of culture. ASC characterization was confirmed in accordance with the International Federation of Adipose Therapeutics and Science/International Society of Cellular Therapy criteria (Bourin et al., 2013).

### 2.6 Generation of hydrogels incubated with released factors by ASCs

**Figure 1:**
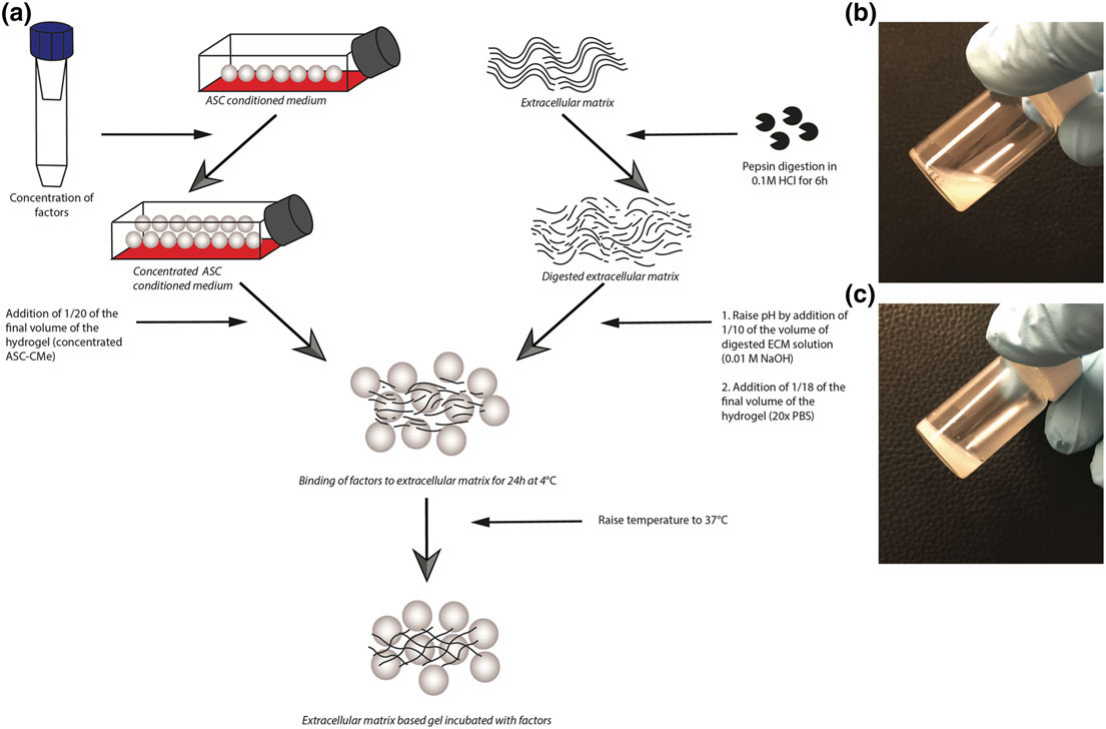
(a) Overview of the generation of ECM‐derived hydrogels incubated with concentrated ASC‐CMe. (b) Pregel solution prior to warming to 37°C. (c) Hydrogel after gelation at 37°C. HCl: hydrogen chloride; NaOH: sodium hydroxide; ASC‐CMe: adipose stromal cell conditioned medium; ECM: extracellular matrix; ASC: adipose stromal cell; PBS: phosphate‐buffered saline [Colour figure can be viewed at wileyonlinelibrary.com]

NAM and DAM samples (*n* = 3) were digested with 2 mg/ml of porcine pepsin (3,200 I.U. Sigma‐Aldrich) in 0.01 M of hydrochloric acid. One milligram of porcine pepsin was used to digest 10 mg of lyophilized acellular matrix. Acellular matrix was digested under constant stirring for 6 hr at RT. Afterwards, the porcine pepsin was inactivated by pH neutralization (pH 7.4) with 0.1 M of sodium hydroxide to reach 0.01 M of final concentration (Figure 1). Then ASC‐CMe was mixed in order to allow released factors to bind to acellular matrix, and finally, salt was added in order to allow for self‐assembly gelation. To maintain the appropriate concentration of acellular matrix in hydrochloric acid to facilitate gelation, ASC‐CMe and PBS were added in a concentrated form. ASC‐CMe was concentrated with the use of 3‐kDa cut‐off Amicon® Ultra‐Centrifugal filters (Sigma‐Aldrich), respectively, 20, 40, and 80 times. Twenty, 40, or 80 times concentrated ASC‐CMe was added and carefully mixed to reach final dilutions of respectively one time (ASC‐CMe1), two times (ASC‐CMe2), and four times (ASC‐CMe4) concentrated ASC‐CMe (Figure 1). The released factors bind to the acellular matrix pregel solutions at 4°C for 24 hr. Next, the pregel solutions were brought to physiological conditions, that is, 1× PBS by adding 20 times concentrated PBS. Finally, the pregel solution was placed in an incubator to allow for self‐assembly gelation for 1 hr at 37°C (Figure 1). Released factors from the incubated hydrogels by ASC‐CMe1, ASC‐CMe2, and ASC‐CMe4 were harvested in serum‐free medium after 24 hr and stored at −80°C for immunoassaying. The respective concentrated ASC‐CMe served as input controls (2.8). Released factors from the incubated hydrogel by ASC‐CMe1 were harvested in serum‐free medium after respectively 24, 48, and 96 hr and stored at −80°C for biological assays. For fibroblast migration and endothelial angiogenesis, the respective concentrated ASC‐CMe1 as well as DMEM serum‐free incubated hydrogels harvested after 24 hr served as controls (2.9 and 2.11). For fibroblast proliferation, the mean cell proliferation of fibroblast after using the respective concentrated ASC‐CMe was set at 100% (2.10). Moreover, hydrogels were not cytotoxic (data not shown).

### 2.7 Characterization of viscoelastic relaxation properties of the hydrogels

NAM and DAM hydrogels (*n* = 3) were formed in 0.8 mm of polydimethylsiloxane rings and placed on a glass slide derived from three different pregel solutions per donor in duplicates (three technical replicates per donor; Table 1). However, due to technical errors, single data were obtained of pregel solution C of donor one and two. Then stress relaxation tests were performed on the NAM and DAM hydrogels with the help of a low‐load compression tester in a nonhydrated environment at RT (Korstgens, Flemming, Wingender, & Borchard, 2001). A stainless steel plunger with a diameter of 0.25 cm was lowered towards the hydrogels at 5 μm/s till the plunger came in contact with the gel (touch load defined as 10 mg). This position was recorded, and similarly, the position of the top of the glass slide was determined. Hydrogel thickness was calculated with the difference of the two positions. Subsequently, the hydrogels were deformed by 20%, and the deformation was held constant for 100 s, and the force response was monitored over time (Peterson, van der Mei, Sjollema, Busscher, & Sharma, 2013; Sharma, Busscher, Terwee, Koopmans, & van Kooten, 2011). Force was converted in stress by dividing with the area of cross section of the plunger, whereas the deformation was converted in strain (deformation/100). The slope of the straight‐line plot between stress and strain during the deformation was taken as stiffness (*E*). Then the strain was held constant at 0.2 for 100 s, and the stress was monitored. Because hydrogels are viscoelastic in nature, the stress did not remain constant but decreased (relaxed) with time. Total relaxation in 100 s was also recorded. Relaxing stiffness (*E*(*t*)) was calculated by dividing relaxing stress with constant strain. The relaxation was understood in terms of a generalized Maxwell model by fitting the experimental data with the equation using the optimization routine in the solver Add‐in of Microsoft Excel 2007. Fitting started with one Maxwell element, and gradually more elements were added till the decrease in chi‐squared (error function) value became insignificant. Each Maxwell element was characterized by the stiffness (*E*_*i*_) and relaxation time constant (*τ*_*i*_) for which it remained active (Figure 5,b–d). The stiffness values were converted into relative importance using the equation *RI*_1_ *=* 100*E_1_/(*E*_1_ + *E*_2_ … *E*_*n*_).

**Table 1:** Nomenclature of nondiabetic acellular matrix and diabetic acellular matrix hydrogels derived from different pregel solutions

	Donor 1	Donor 2	Donor 3
Pregel Solution A	NAM1A/DAM1A	NAM2A/DAM2A	NAM3A/DAM3A
Pregel Solution B	NAM1B/DAM1B	NAM2B/DAM2B	NAM3B/DAM3B
Pregel Solution C	NAM1C/DAM1C	NAM2C/DAM2C	NAM3C/DAM3C

### 2.8 Immunoassay of ECM hydrogels

NAM and DAM hydrogels (*n* = 3) were produced as previously described for the immunoassay (two technical replicates; 2.6). Multiplex immunoassaying (Luminex, R&D systems) was used to measure release of 11 representative ASC‐released proteinaceous factors according to the manufacturer's protocol: VEGF‐A, angiopoetin‐1 (Ang‐1) and angiopoetin‐2 (Ang‐2), matrix metalloproteinase 1 (MMP‐1), tissue inhibitors of metalloproteinase 1 (TIMP‐1), interleukin‐1β (IL‐1β), IL‐6, IL‐8/CXCL8, FGF‐1, hepatocyte growth factor, and monocyte chemotactic protein 1 (MCP‐1/CCL2).

### 2.9 Fibroblast scratch assay

NAM and DAM hydrogels (*n* = 3) were produced as previously described (three technical replicates; 2.6). PK84 fibroblasts were cultured in DMEM (containing 10% FBS, 1% L‐Glut, and 1% P/S). After confluency, PK84 fibroblasts were seeded into a 24‐well plate with a cell density of 17,500 cells per square centimetre. Then cells were serum starved overnight. The next day, a scratch was made with the use of a 1,000 μl of pipette tip. Subsequently, cells were washed with PBS three times, and all the different types of medium were applied. Scratches were analysed after 9 hr.

### 2.10 Fibroblast proliferation assay

NAM and DAM hydrogels (*n* = 3) were produced as previously described (three technical replicates; 2.6). PK84 fibroblasts were cultured in DMEM (containing 10% FBS, 1% L‐Glut, and 1% P/S). After confluency, fibroblasts were seeded into a 96‐well plate with a cell density of 17,500 cells per square centimetre. After 24 hr, the different types of medium were applied for 24 hr (2.6). After 24 hr, 20 μl of MTT (5 mg/ml in PBS) was added and incubated for 3 hr at 37°C. Next, medium was removed, and 100 μl of dimethyl sulfoxide was added to dissolve the formazan crystals. Plates were read at 570 nm. The fibroblast proliferation assay was repeated twice. Results were expressed as percentage of cell proliferation compared with normal culture medium (DMEM containing 10% FBS, 1% L‐Glut, and 1% P/S).

### 2.11 Human umbilical vein endothelial cell sprouting assay

NAM and DAM hydrogels (*n* = 3) were produced as previously described (three technical replicates; 2.6). Instead of DMEM serum free, RPMI serum‐free incubated hydrogels were used as a control (2.6). Human umbilical cord vein endothelial cells (HUVECs) were isolated and cultured in RPMI (containing 10% FBS, 1% L‐Glut, and 1% P/S). Micro Slide Angiogenesis plates (Ibidi GmbH, Germany) were coated with 10 μl of Matrigel™ (BD Bioscience, CA) and incubated at 37°C for 2 hr. HUVECs were resuspended in all different types of medium. Sixty microlitres of medium containing 10,000 cells was added per well. The slides were incubated at 37°C for 6 hr. The sprouting networks that had formed were photographed with the use of an inverted light microscope (Leica Microsystems, DM IL) and the number of loops was counted manually.

### 2.12 Statistics

Scratch assay images were analysed using ImageJ, Version 1.4.3.67 (NIH). Descriptive statistics were used to evaluate the amount of DNA, sGAG, stiffness and factor release profile of the hydrogels, scratch surface area, fibroblast proliferation and the number of loops. Data were expressed as mean with standard error of the mean, except for the controls (serum‐free culture medium and ASC‐CMe1) and stiffness as well as relaxation data of the hydrogels. Data of the controls as well as stiffness and relaxation of the hydrogels were expressed as mean with standard deviation. A one‐tailed *t* test was used to determine the statistical difference between the hydrogels and hydrogels versus controls with the use of Graphpad, Version 7.0c (Graph Pad Software Inc., Los Angeles, CA).

## 3 RESULTS

### 3.1 Fractionation and decellularization of adipose tissue results in an acellular matrix

The fractionation of adipose tissue and its subsequent decellularization process resulted in a completely acellular adipose matrix. Macroscopically, acellular matrix was white of colour, whereas one‐time centrifuged adipose tissue and tSVF were yellow coloured (Figure S1, left panels). Microscopically, the H&E staining together with the absence of perilipin staining showed that the acellular matrix contained neither adipocytes nor other remaining cells. In contrast, the cell number in tSVF was higher as compared with one‐time centrifuged adipose tissue as observed by dense clusters of stromal cells (Figure S1, second series of panels). In accordance with our previous experience, fractionation of adipose tissue disrupted adipocytes, whereas other cell types and ECM were preserved (van Dongen et al., 2016). The absence of perilipin staining confirmed the disruption of adipocytes (Figure S1, third series of panels). Furthermore, Masson's trichrome staining demonstrated visibly higher amounts of collagen (blue) per unit area in acellular matrix as compared with one‐time centrifuged adipose tissue and tSVF (Figure S1, right panels).

### 3.2 Low amount of DNA left in acellular adipose matrix

NAM and DAM samples contained negligible amounts of DNA with 12.83 ± 2.62 ng/mg per dry weight tissue for NAM and 60 ± 53.01 ng/mg per dry weight tissue for DAM (*p* > 0.05; Figure S2A). The amount of DNA in DAM is, however, higher than the amount of DNA in NAM. All samples, except for one DAM sample, contained a lower amount of DNA than 50 ng/mg per dry weight tissue, which is the standard for successful decellularization. DAPI staining could not reveal nuclei in both NAM as well as DAM as compared with the high number of nuclei after the fractionation of adipose tissue procedure (Figure S2B).

### 3.3 No difference in sulphated glycosaminoglycans

There was no difference in amount of sGAGs in NAM and DAM samples with 0.93 ± 0.31 and 0.69 ± 0.31 μg sGAG per milligram dry weight ECM respectively (*p* > 0.05; Figure S3). This was confirmed by Alcian blue staining of control adipose tissue, tSVF, and as ECM samples: Here, similar levels of staining were detected (Figure S3B).

### 3.4 Viscoelastic relaxation properties of NAM hydrogels

**Figure 2:**
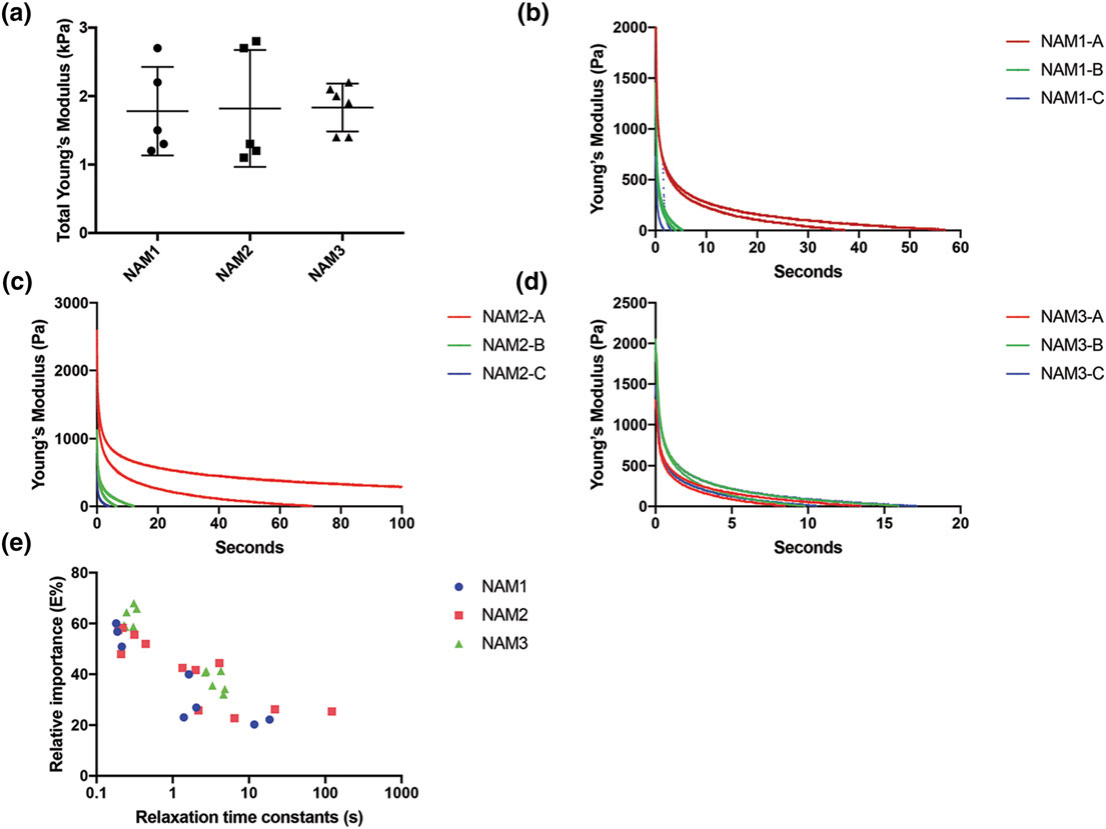
(a) The stiffness (kPa) for only NAM hydrogels (*n* = 3). DAM hydrogels collapsed and could not be evaluated. (b–d) Decrease in stiffness of NAM hydrogels displayed as a function of time. All replicates of each donor (*n* = 3) are presented. (*E*) Relative importance of individual Maxwell elements as a function of relative time constants in seconds for each replicate of all donors (*n* = 3). Each point represents one Maxwell element. NAM: nondiabetic acellular matrix; DAM: diabetic acellular matrix; kPa: kiloPascal [Colour figure can be viewed at wileyonlinelibrary.com]

Proteolytic treatment at RT of NAM and DAM liquified these matrices, which formed stable hydrogels upon warming to 37°C, albeit of relatively low mechanical strength. Viscoelastic relaxation properties were determined for all NAM and DAM hydrogels; however, all DAM hydrogels and one NAM gel collapsed during the measurements and yielded no data. This indicates that structural differences are present in ECM derived from diabetic donors in comparison with ECM derived from nondiabetic donors. Average stiffness for all measured NAM hydrogels was relatively low with 1.81 ± 0.02 kPa (Figure 2a). For each donor, three independent pregel solutions derived from the same donor and ECM isolation were produced by pepsin digestion (Table 1). Different pregel solutions showed a large intradonor variation of stiffness (Figure 2a). The large intradonor variation of stiffness warrants standardization of the gelation procedure. The intradonor variation was also present in the relaxation properties of the measured NAM hydrogels. Relaxation of stiffness showed a fast decrease with the stiffness reaching zero within about 20 s (Figure 2b–d). This was typical for most of the replicates, that is, NAM1, −2 and −3 with each requiring 2 to 3 (Table 1 and Figure 2e). The fast decrease shows that adipose tissue‐derived ECM hydrogels are much more viscous than elastic. A more viscous gel is less resistant to mechanical stress, that is, more prone to collapse and therefore less suitable for clinical applications, which relate to movement and friction such as wound healing. Stress relaxation as a function of time was measured with the use of a Maxwell model with each element in this model having a spring constant related to the elastic part of the gel and a relaxation time constant representing a viscous part of the gel. Two or three elements were sufficient and the addition of more elements did not result in an improved quality of the fit. The first element having a high relative importance and a relaxation time constant of less than 1 s was most likely liquid that was pushed out of the gel (Figure 2). The second element with a relaxation time constant between 1 and 10 s, which was most likely ECM. Interestingly, in some hydrogels, a third element was shown with a relaxation time constant between 10 and 100 s. Hydrogels that present a third element were also the hydrogels with the highest stiffness and lowest relaxation.

### 3.5 Hydrogels bind and release ASC‐secreted factors

**Figure 3:**
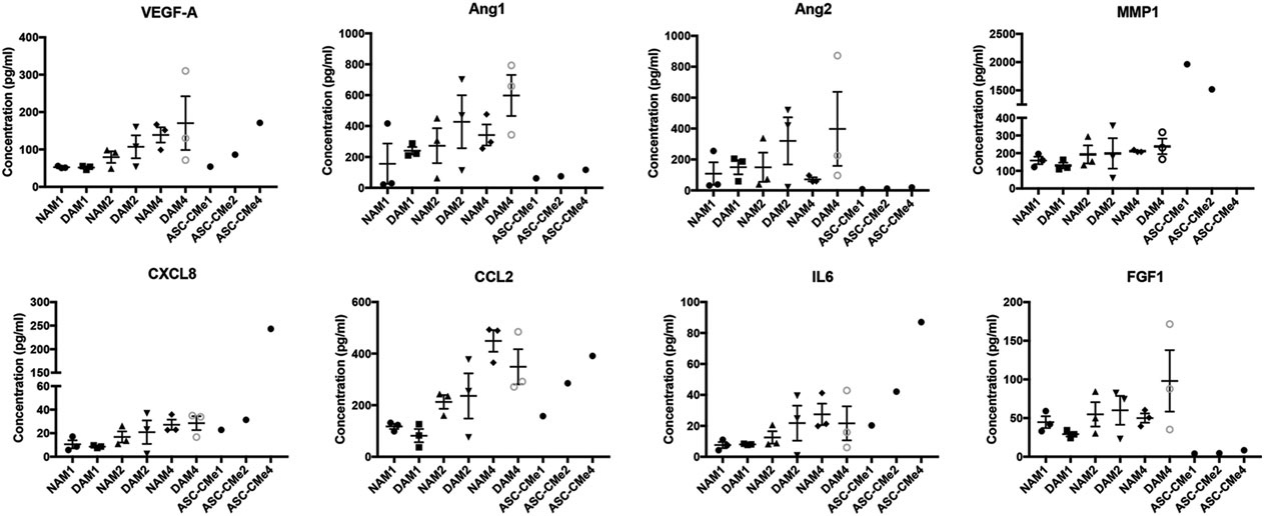
Statistical analyses of the concentration of released factors in baseline ASC‐CMe and conditioned medium derived from NAM and DAM samples (*n* = 3). ASC‐CMe: adipose derived stromal cell conditioned medium; NAM: nondiabetic acellular matrix; DAM: diabetic acellular matrix. One, two, and four displays the concentration factor of the factors in ASC‐CMe as well as the concentration of the hydrogel released factors injected in NAM and DAM samples. The concentration of MMP1 exceeded the upper limit of the assay in ASC‐CMe4 and was excluded. Results are expressed as mean with standard error of the mean

Hydrogels derived from NAM and DAM samples mixed with concentrated CMe from cultured ASC bind released factors from ASC and had released part of the measured factors after 24 hr. The concentrations of factors were lower in the conditioned medium derived from NAM and DAM samples in comparison with the three baseline concentrations of ASC‐CMe for MMP‐1, CXCL8, and IL‐6. The difference in concentration of factors indicates that the release of bound factors extends well beyond 24 hr. For Ang‐1, Ang‐2, and FGF‐1, a higher concentration in conditioned medium derived from hydrogels as compared with the baseline concentrations was found. This implies that the hydrogels release more factors than initially were mixed in with ASC‐CMe. This indicates that there were still factors present in ECM after decellularization that were detectable by the immunoassay, albeit that their bioactivity might not be retained after the harsh decellularization procedures. There was no significant difference in release pattern of any of the measured factors between nondiabetic and diabetic‐derived hydrogels (*p* > 0.05; Figure 3). The concentrations of IL‐1β and hepatocyte growth factor were below the detection limit. In contrast, TIMP‐1 concentrations exceeded the maximal detection limit and were therefore not plotted. These high concentrations of TIMP‐1 indicate that most of the MMP‐1 released by ASC and, therefore, released by the hydrogels is likely rendered inactive by binding of TIMP‐1. Moreover, large interdonor variations in releasing patterns were observed, especially in diabetic‐derived hydrogels and mainly when two and four times concentrated ASC‐CMe was mixed with the gels. This indicates a donor dependent binding capacity limitation of specific factors such as VEGF‐A, Ang‐1, Ang‐2, MMP‐1, and CCL2.

### 3.6 Similar PK84 fibroblast migration when treated with released factors by CMe‐loaded NAM and DAM hydrogels

**Figure 4:**
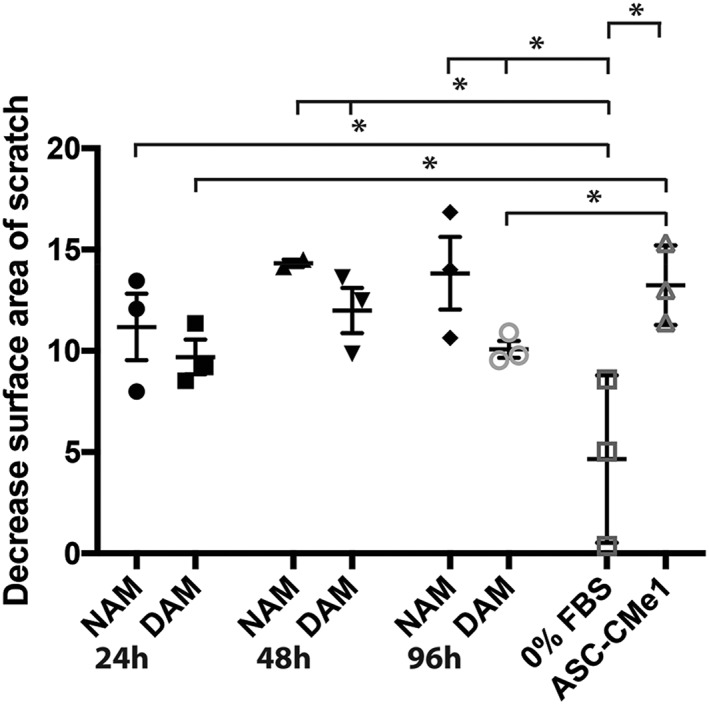
Statistical analyses of changes of the scratch surface area (9 hr) compared with controls (serum‐free culture medium and ASC‐CMe1) for conditioned medium derived from NAM and DAM hydrogels (*n* = 3) with released factors after 24, 48, and 96 hr. NAM: nondiabetic acellular matrix; DAM: diabetic acellular matrix; ASC‐CMe1: one time concentrated (undiluted) adipose‐derived stromal cell conditioned medium; FBS: fetal bovine serum. *Significant smaller scratch surface area visible after the use of NAM 24hr, NAM 48hr, and NAM 96hr as well as DAM 48hr and DAM 96hr in comparison with serum‐free culture medium (*p* < 0.05). Moreover, the use of ASC‐CMe1 reduced the scratch area in comparison with serum‐free culture medium (*p* < 0.05). Significant smaller scratch area visible after the use of ASC‐CMe1 in comparison with DAM 24hr and DAM 96hr (*p* < 0.05). Results are expressed as mean with standard error of the mean for NAM and DAM hydrogels. Results are expressed as mean with standard deviation for serum‐free culture medium and ASC‐CMe1

In comparison with serum‐free controls, conditioned medium from ASC (ASC‐CMe1) promoted closure of damaged fibroblast monolayers (Figure 4). Similarly, factors released from either NAM or DAM gels promoted closure even after 96 hr of release (Figure 4). A similar migration speed was detected between fibroblasts treated with factors from CMe‐loaded NAM and DAM hydrogels released after 24, 48, and 96 hr (*p* > 0.05; Figure 4). However, in comparison with ASC‐CMe1, factors from CMe‐loaded DAM hydrogels released after 24 and 96 hr showed a smaller decrease in scratch surface area (*p* < 0.05). For the NAM groups, all groups showed a larger decrease in scratch surface area in comparison with controls, that is, serum‐free culture medium (*p* < 0.05). For the DAM group, released factors from CMe‐loaded hydrogels after 48 and 96 hr in comparison with serum‐free controls showed a larger decrease in scratch surface area (*p* < 0.05). This suggests that acellular adipose tissue matrix hydrogels released factors for at least 96 hr as observed by stimulated migration of fibroblasts.

### 3.7 Proliferation of PK84 fibroblasts is increased with released factors by CMe‐loaded DAM hydrogels

**Figure 5:**
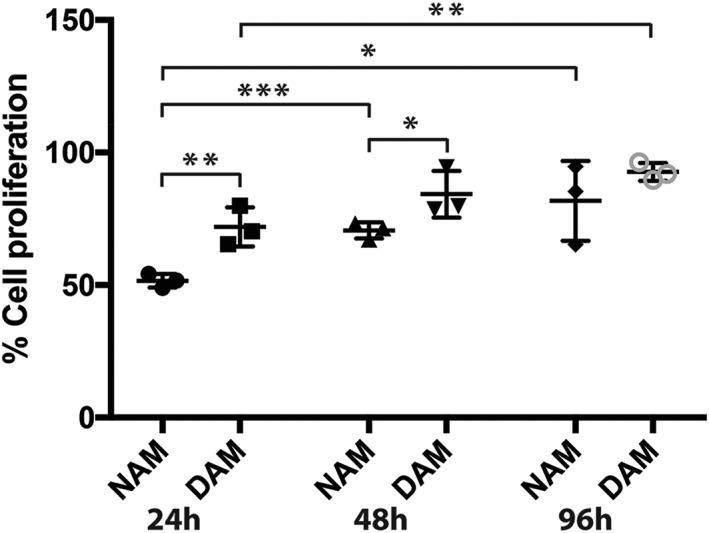
Statistical analyses of fraction of proliferating cells compared with ASC‐CMe for conditioned medium derived from NAM and DAM hydrogels (*n* = 3) with released factors after 24, 48, and 96 hr. NAM: nondiabetic acellular matrix; DAM: diabetic acellular matrix; ASC‐CMe1: one time concentrated (undiluted) adipose derived stromal cell conditioned medium. *Significantly more proliferation occurred between NAM 24hr versus NAM 96hr and NAM 48hr versus DAM 48hr (*p* < 0.05). **Significantly more proliferation occurred between NAM 24hr versus DAM 24hr and DAM 24hr versus DAM 96hr (*p* < 0.01). ***Significantly more proliferation occurred between NAM 24hr versus NAM 48hr (*p* < 0.001). Results are expressed as mean with standard error of the mean for NAM and DAM hydrogels

Factors released from both NAM and DAM gels promoted proliferation for 96 hr (Figure 5). Proliferation was higher in the DAM group as compared with the NAM group with factors being released for 24 and 48 hr (*p* < 0.01 and *p* < 0.05, respectively; Figure 5). Furthermore, the proliferation was increased in the NAM group with released factors after 24 versus 48 hr (*p* < 0.001) as well as after 24 versus 96 hr (*p* < 0.05) and was higher in the DAM group after 24 versus 96 hr (*p* < 0.01).

### 3.8 Similar sprouting ability of HUVEC when treated with released factors by CMe‐loaded NAM and DAM hydrogels

**Figure 6:**
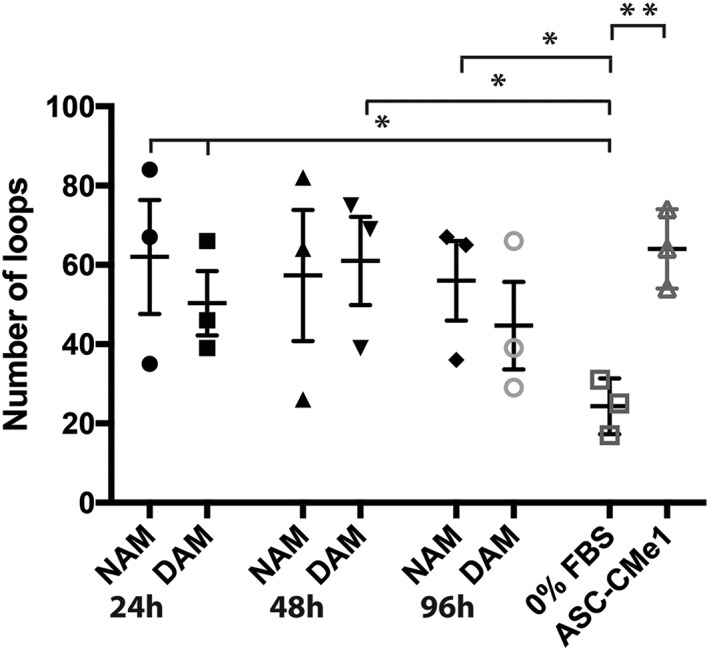
Statistical analyses of the number of loops of the 6‐hr sprouting assay compared with controls (serum‐free culture medium and ASC‐CMe1) for conditioned medium derived from NAM and DAM hydrogels (*n*=3) with released factors after 24, 48, and 96 hr. NAM: nondiabetic acellular matrix; DAM: diabetic acellular matrix; ASC‐CMe1: one time concentrated (undiluted) adipose‐derived stromal cell conditioned medium; FBS: fetal bovine serum. *Significantly more loops visible after the use of NAM24h, NAM96h, DAM24h as well as DAM48h in comparison with serum free culture medium (p < 0.05). **Significantly more loops visible after the use of ASC‐CMe1 in comparison with serum free culture medium (*p* < 0.01). Results are expressed as mean with standard error of the mean for NAM and DAM hydrogels. Results are expressed as mean with standard deviation for serum‐free culture medium and ASC‐CMe1

Sprouting of HUVEC was similar after treatment with factors released from CMe‐loaded NAM hydrogels compared with DAM hydrogels (*p* > 0.05; Figure 6). Moreover, a comparison of the total number of loops between different times, that is, 24, 48, and 96 hr of released factors was similar for both types of hydrogels (*p* > 0.05; Figure 6). For the NAM groups, released factors from CMe‐loaded hydrogels after 24 hr as well as 96 hr in comparison with serum‐free controls showed more loops (*p* < 0.05). Unexpectedly, no significant difference in the group of released factors from CMe‐loaded NAM hydrogels after 48 hr compared with serum‐free medium was found. This is probably caused by large interdonor variation, because the mean number of loops is higher in the group of released factors from CMe‐loaded NAM hydrogels after 48 hr. Moreover, these results indicate that CMe‐loaded hydrogels function as a controlled slow release scaffold of factors that stimulate sprouting even after 96 hr. For the DAM group, released factors from CMe‐loaded hydrogels after 24 hr as well as 48 hr in comparison with serum‐free culture medium showed more loops (*p* < 0.05). The mean number of loops for ASC‐CMe1 was comparable with the mean number of loops for the NAM and DAM hydrogels (*p* > 0.05).

## 4 DISCUSSION

In this study, we demonstrated that acellular ECM hydrogels from decellularized adipose tissue incubated with factors released by ASC functioned as a controlled slow release scaffold. These ECM hydrogels bound a series of different factors, which were released in an incremental fashion for at least 96 hr and maintained their biological activity. These ECM hydrogels showed to be noncytotoxic as well. Diabetic origin of the hydrogels did not substantially affect the biological activity nor concentrations of the released factors; small differences in kinetics of biological assays were seen for unknown reasons. Yet diabetic ECM‐derived hydrogels had a too low mechanical strength and are therefore less suitable for clinical applications. Thus, CMe‐incubated nondiabetic‐derived hydrogels seems to be a promising new treatment modality to augment wound healing.

The released factors by ASC‐CMe‐loaded ECM hydrogels stimulated several important wound healing‐related processes including an increased fibroblast proliferation and migration as well as sprouting by HUVECs, that is, surrogate angiogenesis. Diabetic origin did not affect either of these processes, except that cell proliferation of fibroblasts was reduced 24 and 48 hr after release of factors by nondiabetic hydrogels in comparison with diabetic hydrogels. Yet the immunoassay showed similar concentrations of factors released by both types of hydrogels. This suggests that the releasing pattern of some factors might be different when released by hydrogels of diabetic origin, for example, a faster release. A faster release of factors results, however, in a faster depletion of factors in ASC‐CMe‐loaded hydrogels and thus a shorter time to promote wound healing. Moreover, factors will be actively subtracted from the hydrogel in vivo instead of accumulation of factors until a concentration balance is reached between inside and outside the hydrogel. Additionally, fibroblast cell proliferation increased over time after using released factors in an incremental fashion up to 96 hr.

Wound healing comprises different processes that show spatiotemporal overlapping phases with different processes of which some, for example, angiogenesis, fibroblast proliferation, and migration are investigated in this study. Our results suggest that released factors from ASC‐CMe‐loaded hydrogels influence both the course and kinetics of these phases. These human ECM‐derived hydrogels allow the allogenic administration because both factors released by ASC as well as ECM will not induce an adverse immune reaction. In fact, ECM components are evolutionary highly conserved, which warrants the use of animal‐derived ECM hydrogels, for example, pig dermal ECM hydrogels. The potential therapeutic value of this “one‐donor‐for‐all” treatment modality for wound healing purposes is based on biological properties as well as physical properties. Biologically, a plethora of factors can be bound and released by ECM. In this way, wound healing can be enhanced by influencing processes like angiogenesis, apoptosis, chemoattraction of immune cells, and support of local mesenchymal or progenitor cells (Barrientos, Stojadinovic, Golinko, Brem, & Tomic‐Canic, 2008). Angiogenesis is enhanced by VEGF and FGF‐1, especially under hypoxia (Rehman et al., 2004). ASC‐CMe, which contains VEGF and FGF‐1, increases endothelial proliferation and suppresses cell apoptosis under hypoxia. Both proliferation of endothelial cells and survival are important mechanisms in wound healing (Chavakis & Dimmeler, 2002). Additionally, VEGF also functions as a chemoattractant for local progenitor cells as well as macrophages (Chen, Tredget, Wu, & Wu, 2008). Macrophages, attracted by chemokines such as ASC‐released CXCL8, play an important role in the different phases of wound healing (Weidenbusch & Anders, 2012). During the inflammation phase, macrophages phagocytose pathogens and cellular debris. During the proliferation phase, epithelial proliferation is enhanced by activated macrophages (Weidenbusch & Anders, 2012). Furthermore, re‐epithelialization by keratinocyte proliferation and migration is also stimulated by CXCL8, which was bound and released by our ECM hydrogels (Barrientos et al., 2008). Besides increased re‐epithelialization, CXCL8 is also responsible for the attraction of leukocytes, which results in an increased phagocytosis during the inflammation phase (Rennekampff et al., 2000). The presence of leukocytes and macrophages is prolonged by the presence of MCP1, as MCP1 is a chemoattractant for macrophages, mast cells, and T‐cells (Barrientos et al., 2008). A prolonged presence of leukocytes and macrophages results in a sustained proinflammatory state of the wound (Wetzler, Kampfer, Stallmeyer, Pfeilschifter, & Frank, 2000). After the inflammation phase, a decrease in inflammatory signalling is needed to induce formation of granulation tissue and subsequently re‐epithelialization. In vitro and in vivo experiments have shown that GAG‐binding sites are able to modulate chemokine gradients of MCP1 and IL8 by subduction in excisional wounds (Lohmann et al., 2017). In this way, ECM hydrogels incubated with ASC‐CMe can modulate inflammation in wounds by both releasing and attracting growth factors and chemokines.

Besides the biological function of our studied hydrogel, its physical properties are also of crucial importance. Physical properties of the hydrogel such as stiffness affect ASC and other cells present in the surrounding tissue of the wound. Low stiffness hydrogels promote adipogenic differentiation of native ASC, whereas stiff hydrogels promote their osteogenic differentiation (Schellenberg et al., 2014). The soft nondiabetic hydrogels support adipogenic differentiation of host tissues ASC; however, the bound factors together with the stiffness of the hydrogel dictate final cell fate (Schellenberg et al., 2014). A soft hydrogel is also easier infiltrated by host cells like ASC and endothelial cells than stiff hydrogels. In this way, both ASC and endothelial cells can stimulate angiogenesis inside the hydrogel, which might result in increased wound healing rates (Terlizzi, Kolibabka, Burgess, Hammes, & Harmsen, 2017). It remains to be determined whether the stability and longevity of these hydrogels supports cells' maintenance, migration, and differentiation. Also, it is unknown if the hydrogels with a low stability can resist all mechanical stress during application of the hydrogel in vivo. The enzymatic degradation to convert complex ECM structures into gels has inspired physical chemists to compile prediction models (Abete, de Candia, Lairez, & Coniglio, 2004; Jones, Liang, Lin, Jiao, & Sun, 2014). However, from a pragmatic point of view, production of (pre)gels from freeze‐dried, fine‐powdered ECMs requires careful standardization of enzyme treatment. These should consider, for example, the disease background of the ECM, because our results show differences between diabetic and nondiabetic ECM hydrogels, that is, gels of diabetic origin collapse. Regarding future application, diabetic hydrogels will not suffice due to their limited physical strength. As a matter of fact, viscoelasticity measurements proved impossible because diabetic hydrogels collapsed readily. This indicates the presence of structural differences in matrix between nondiabetic and diabetic donors. This difference could be caused by accumulation of AGEs in ECM, which results in more cross links between proteins and therefore a high stability of the ECM proteins (Voziyan et al., 2014). The major component of ECM is Collagen Type I, and glucose‐based AGEs may cause a high level of intramolecular Lys‐Arg and Lys‐Lys cross links that increase the molecule's stiffness (Gautieri, Redaelli, Buehler, & Vesentini, 2014). The glycation and cross linking masks potential pepsin digestion sites and thus yields different, likely larger, fragments upon pepsin treatment. As it appeared, these fragments did not yield hydrogels from diabetic ECM. Instead of adipose tissue, other sources of matrices with a higher stiffness might also suit to enhance wound healing. Our recent data show that ECM hydrogels from heart or aorta tissue have a higher viscoelasticity (to be published elsewhere).

To date, most studies that evaluate decellularized adipose tissue focus on tissue engineering of a new subcutaneous fat layer rather than its use to augment wound healing (Choi et al., 2010; Choi et al., 2011; Debels et al., 2017; Flynn, 2010; Uriel et al., 2008). Moreover, all these studies used adipose matrix‐derived hydrogels without incubation of paracrine factors. Without the use of additional paracrine factors and cytokines, the soft adipose matrix will most likely only regenerate a soft subcutaneous fat layer. Yet, to regenerate a dermal layer for wound healing purposes, the addition of factors released by ASC is warranted. A combination of factors released by ASC and a soft adipose matrix‐derived hydrogel might, therefore, be an ideal treatment modality for full thickness wounds.

Both types of gel released functional factors over a longer time. However, a large interdonor variation remains regardless the origin of the ECM. The interdonor variation might be caused by the fact that ECM derived from different donors with different “pack years” of diabetes. In this way, the amount of AGEs accumulation is different between donors. Moreover, liposuction procedures are performed on lean patients and patients with obesity. However, obese patients frequently acquire DM type 2 with high levels of glucose prior to diagnosis. It is feasible that ECM derived from nondiabetic donors was exposed to high levels of glucose already. Finally, the medical history of the anonymous donations was unknown. We cannot exclude the influence of age, gender, BMI, and other confounding factors on the donor variation (Di Taranto et al., 2015; Dos‐Anjos Vilaboa, Navarro‐Palou, & Llull, 2014; Engels et al., 2013; Maredziak, Marycz, Tomaszewski, Kornicka, & Henry, 2016). This warrant to standardize the generation of factor‐loaded ECM hydrogels with regard to both producing ASC and to source of the ECM. The low yield of adipose‐derived ECM (*w*/*v* base) hampers clinical application of adipose tissue‐derived hydrogels.

In summary, this study shows that several important wound healing‐related processes, that is, angiogenesis, fibroblast migration, and proliferation are stimulated by a sustained release of growth factors from ECM‐derived hydrogels. Therefore, the use adipose tissue ECM‐derived hydrogels incubated with released factors from ASCs become a promising new treatment modality to augment disturbed dermal wound healing, for example, diabetic ulcers. Animal studies are warranted as an essential prelude to clinical trials.

## FUNDING INFORMATION

This study was funded by the University Medical Center Groningen.

## CONFLICT OF INTEREST

The authors have no conflict of interest to disclose in relation to the content of this work.

## AUTHOR CONTRIBUTIONS

J. A. vD., H. P. S., B. vdL., and M. C. H. contributed to the design of the manuscript. All experiments were performed by J. A. vD. in collaboration with V. G., L. A. B., and G. R. L. P. S. has made substantial contribution to the viscoelastic experiments by designing and analysing the experiments. H. P. S. and B. vdL. performed the liposuction. J. A. vD. analysed all experiments. All authors have critically reviewed the manuscript for important intellectual content.

## Supporting information


**Fig. S1** Light micrographs of hematoxylin & eosin staining, perilipin A staining and Masson's trichrome staining of respectively centrifuged adipose tissue, tSVF and extracellular matrix. tSVF = tissue stromal vascular fraction.

**Fig. S2** **(A)** Statistical analyses of DNA contents per dry weight ECM (ng/mg) of NAM and DAM samples (*n* = 3). **(B)** Immunofluorescent microscope photographs of DAPI staining. ECM = extracellular matrix, NAM = non‐diabetic acellular matrix, DAM = diabetic acellular matrix, AT = adipose tissue, tSVF = tissue stromal vascular fraction, NS = non‐significant. Results are expressed as mean with standard error of the mean.

**Fig. S3** **(A)** Statistical analyses of sGAG contents per dry weight ECM (μg/mg) of NAM and DAM samples (*n* = 3). **(B)** Light microscope photographs of alcian blue staining. sGAG = sulphated glycosaminoglycan, ECM = extracellular matrix, NAM = non‐diabetic acellular matrix, DAM = diabetic acellular matrix, AT = adipose tissue, tSVF = tissue stromal vascular fraction, NS = non‐significant. Results are expressed as mean with standard error of the mean.

## References

[term2843-bib-0001] Abete, T. , de Candia, A. , Lairez, D. , & Coniglio, A. (2004). Percolation model for enzyme gel degradation. Physical Review Letters, 93(22), 228301 10.1103/PhysRevLett.93.228301 15601123

[term2843-bib-0002] Barrientos, S. , Stojadinovic, O. , Golinko, M. S. , Brem, H. , & Tomic‐Canic, M. (2008). Growth factors and cytokines in wound healing. Wound Repair and Regeneration, 16(5), 585–601. 10.1111/j.1524-475X.2008.00410.x 19128254

[term2843-bib-0003] Bourin, P. , Bunnell, B. A. , Casteilla, L. , Dominici, M. , Katz, A. J. , March, K. L. , … Gimble, J. M. (2013). Stromal cells from the adipose tissue‐derived stromal vascular fraction and culture expanded adipose tissue‐derived stromal/stem cells: a joint statement of the International Federation for Adipose Therapeutics and Science (IFATS) and the International Society for Cellular Therapy (ISCT). Cytotherapy, 15(6), 641–648. 10.1016/j.jcyt.2013.02.006 23570660 PMC3979435

[term2843-bib-0004] Cervelli, V. , De Angelis, B. , Lucarini, L. , Spallone, D. , Balzani, A. , Palla, L. , … Cerulli, P. (2010). Tissue regeneration in loss of substance on the lower limbs through use of platelet‐rich plasma, stem cells from adipose tissue, and hyaluronic acid. Advances in Skin & Wound Care, 23(6), 262–272. 10.1097/01.ASW.0000363551.82058.36 20489388

[term2843-bib-0005] Chavakis, E. , & Dimmeler, S. (2002). Regulation of endothelial cell survival and apoptosis during angiogenesis. Arteriosclerosis, Thrombosis, and Vascular Biology, 22(6), 887–893. 10.1161/01.ATV.0000017728.55907.A9 12067894

[term2843-bib-0006] Chen, L. , Tredget, E. E. , Wu, P. Y. , & Wu, Y. (2008). Paracrine factors of mesenchymal stem cells recruit macrophages and endothelial lineage cells and enhance wound healing. PLoS ONE, 3(4), e1886 10.1371/journal.pone.0001886 18382669 PMC2270908

[term2843-bib-0007] Choi, J. S. , Kim, B. S. , Kim, J. Y. , Kim, J. D. , Choi, Y. C. , Yang, H. J. , … Cho, Y. W. (2011). Decellularized extracellular matrix derived from human adipose tissue as a potential scaffold for allograft tissue engineering. Journal of Biomedical Materials Research. Part A, 97(3), 292–299. 10.1002/jbm.a.33056 21448993

[term2843-bib-0008] Choi, J. S. , Yang, H. J. , Kim, B. S. , Kim, J. D. , Lee, S. H. , Lee, E. K. , … Lee, H. Y. (2010). Fabrication of porous extracellular matrix scaffolds from human adipose tissue. Tissue Engineering. Part C, Methods, 16(3), 387–396. 10.1089/ten.tec.2009.0276 19601696

[term2843-bib-0009] Corselli, M. , Chen, C. W. , Sun, B. , Yap, S. , Rubin, J. P. , & Peault, B. (2012). The tunica adventitia of human arteries and veins as a source of mesenchymal stem cells. Stem Cells and Development, 21(8), 1299–1308. 10.1089/scd.2011.0200 21861688 PMC3353742

[term2843-bib-0010] Crapo, P. M. , Gilbert, T. W. , & Badylak, S. F. (2011). An overview of tissue and whole organ decellularization processes. Biomaterials, 32(12), 3233–3243. 10.1016/j.biomaterials.2011.01.057 21296410 PMC3084613

[term2843-bib-0011] Debels, H. , Gerrand, Y. W. , Poon, C. J. , Abberton, K. M. , Morrison, W. A. , & Mitchell, G. M. (2017). An adipogenic gel for surgical reconstruction of the subcutaneous fat layer in a rat model. Journal of Tissue Engineering and Regenerative Medicine, 11(4), 1230–1241. 10.1002/term.2025 25950280

[term2843-bib-0012] Di Taranto, G. , Cicione, C. , Visconti, G. , Isgro, M. A. , Barba, M. , Di Stasio, E. , … Lattanzi, W. (2015). Qualitative and quantitative differences of adipose‐derived stromal cells from superficial and deep subcutaneous lipoaspirates: A matter of fat. Cytotherapy, 17(8), 1076–1089. 10.1016/j.jcyt.2015.04.004 26002819

[term2843-bib-0013] Dos‐Anjos Vilaboa, S. , Navarro‐Palou, M. , & Llull, R. (2014). Age influence on stromal vascular fraction cell yield obtained from human lipoaspirates. Cytotherapy, 16(8), 1092–1097. 10.1016/j.jcyt.2014.02.007 24726656

[term2843-bib-0014] Engels, P. E. , Tremp, M. , Kingham, P. J. , di Summa, P. G. , Largo, R. D. , Schaefer, D. J. , & Kalbermatten, D. F. (2013). Harvest site influences the growth properties of adipose derived stem cells. Cytotechnology, 65(3), 437–445. 10.1007/s10616-012-9498-2 23095943 PMC3597178

[term2843-bib-0015] Farndale, R. W. , Sayers, C. A. , & Barrett, A. J. (1982). A direct spectrophotometric microassay for sulfated glycosaminoglycans in cartilage cultures. Connective Tissue Research, 9(4), 247–248. 10.3109/03008208209160269 6215207

[term2843-bib-0016] Flynn, L. E. (2010). The use of decellularized adipose tissue to provide an inductive microenvironment for the adipogenic differentiation of human adipose‐derived stem cells. Biomaterials, 31(17), 4715–4724. 10.1016/j.biomaterials.2010.02.046 20304481

[term2843-bib-0017] Gautieri, A. , Redaelli, A. , Buehler, M. J. , & Vesentini, S. (2014). Age‐ and diabetes‐related nonenzymatic crosslinks in collagen fibrils: Candidate amino acids involved in Advanced Glycation End‐products. Matrix Biology, 34, 89–95. 10.1016/j.matbio.2013.09.004 24060753

[term2843-bib-0018] Han, S. K. , Kim, H. R. , & Kim, W. K. (2010). The treatment of diabetic foot ulcers with uncultured, processed lipoaspirate cells: A pilot study. Wound Repair and Regeneration, 18(4), 342–348. 10.1111/j.1524-475X.2010.00593.x 20492632

[term2843-bib-0019] Haucke, E. , Navarrete‐Santos, A. , Simm, A. , Silber, R. E. , & Hofmann, B. (2014). Glycation of extracellular matrix proteins impairs migration of immune cells. Wound Repair and Regeneration, 22(2), 239–245. 10.1111/wrr.12144 24635174

[term2843-bib-0020] Jaspers, M. E. , Brouwer, K. M. , van Trier, A. J. , Groot, M. L. , Middelkoop, E. , & van Zuijlen, P. P. (2017). Effectiveness of autologous fat grafting in adherent scars: Results obtained by a comprehensive scar evaluation protocol. Plastic and Reconstructive Surgery, 139(1), 212–219. 10.1097/PRS.0000000000002891 27632398

[term2843-bib-0021] Jones, C. A. , Liang, L. , Lin, D. , Jiao, Y. , & Sun, B. (2014). The spatial‐temporal characteristics of type I collagen‐based extracellular matrix. Soft Matter, 10(44), 8855–8863. 10.1039/C4SM01772B 25287650

[term2843-bib-0022] Korstgens, V. , Flemming, H. C. , Wingender, J. , & Borchard, W. (2001). Uniaxial compression measurement device for investigation of the mechanical stability of biofilms. Journal of Microbiological Methods, 46(1), 9–17. 10.1016/S0167-7012(01)00248-2 11412909

[term2843-bib-0023] Lin, G. , Garcia, M. , Ning, H. , Banie, L. , Guo, Y. L. , Lue, T. F. , & Lin, C. S. (2008). Defining stem and progenitor cells within adipose tissue. Stem Cells and Development, 17(6), 1053–1063. 10.1089/scd.2008.0117 18597617 PMC2865901

[term2843-bib-0024] Lohmann, N. , Schirmer, L. , Atallah, P. , Wandel, E. , Ferrer, R. A. , Werner, C. , … Freudenberg, U. (2017). Glycosaminoglycan‐based hydrogels capture inflammatory chemokines and rescue defective wound healing in mice. Science Translational Medicine, 9(386). 10.1126/scitranslmed.aai9044 28424334

[term2843-bib-0025] Maredziak, M. , Marycz, K. , Tomaszewski, K. A. , Kornicka, K. , & Henry, B. M. (2016). The influence of aging on the regenerative potential of human adipose derived mesenchymal stem cells. Stem Cells International, 2016, 2152435. 26941800 10.1155/2016/2152435 PMC4749808

[term2843-bib-0026] Mescher, L. A. (2010). Junquira's basic histology. Text and atlas The McGraw‐Hill Companies.

[term2843-bib-0027] Mott, J. D. , Khalifah, R. G. , Nagase, H. , Shield, C. F. 3rd , Hudson, J. K. , & Hudson, B. G. (1997). Nonenzymatic glycation of type IV collagen and matrix metalloproteinase susceptibility. Kidney International, 52(5), 1302–1312. 10.1038/ki.1997.455 9350653

[term2843-bib-0028] Negenborn, V. L. , Groen, J. W. , Smit, J. M. , Niessen, F. B. , & Mullender, M. G. (2016). The use of autologous fat grafting for treatment of scar tissue and scar‐related conditions: A systematic review. Plastic and Reconstructive Surgery, 137(1), 31e–43e. 10.1097/PRS.0000000000001850 26710059

[term2843-bib-0029] Peng, Z. , Yang, X. , Qin, J. , Ye, K. , Wang, X. , Shi, H. , … Lu, X. (2017). Glyoxalase‐1 overexpression reverses defective proangiogenic function of diabetic adipose‐derived stem cells in streptozotocin‐induced diabetic mice model of critical limb ischemia. Stem Cells Translational Medicine, 6(1), 261–271. 10.5966/sctm.2015-0380 28170200 PMC5442730

[term2843-bib-0030] Peterson, B. W. , van der Mei, H. C. , Sjollema, J. , Busscher, H. J. , & Sharma, P. K. (2013). A distinguishable role of eDNA in the viscoelastic relaxation of biofilms. MBio, 4(5), e00497–e00413. 10.1128/mBio.00497-13 24129256 PMC3812712

[term2843-bib-0031] Pozzi, A. , Zent, R. , Chetyrkin, S. , Borza, C. , Bulus, N. , Chuang, P. , … Voziyan, P. (2009). Modification of collagen IV by glucose or methylglyoxal alters distinct mesangial cell functions. Journal of the American Society of Nephrology, 20(10), 2119–2125. 10.1681/ASN.2008080900 19608705 PMC2754111

[term2843-bib-0032] Rehman, J. , Traktuev, D. , Li, J. , Merfeld‐Clauss, S. , Temm‐Grove, C. J. , Bovenkerk, J. E. , … March, K. L. (2004). Secretion of angiogenic and antiapoptotic factors by human adipose stromal cells. Circulation, 109(10), 1292–1298. 10.1161/01.CIR.0000121425.42966.F1 14993122

[term2843-bib-0033] Rennekampff, H. O. , Hansbrough, J. F. , Kiessig, V. , Dore, C. , Sticherling, M. , & Schroder, J. M. (2000). Bioactive interleukin‐8 is expressed in wounds and enhances wound healing. The Journal of Surgical Research, 93(1), 41–54. 10.1006/jsre.2000.5892 10945942

[term2843-bib-0034] Roehm, K. D. , Hornberger, J. , & Madihally, S. V. (2016). In vitro characterization of acelluar porcine adipose tissue matrix for use as a tissue regenerative scaffold. Journal of Biomedical Materials Research. Part A, 104(12), 3127–3136. 10.1002/jbm.a.35844 27465789

[term2843-bib-0035] Schellenberg, A. , Joussen, S. , Moser, K. , Hampe, N. , Hersch, N. , Hemeda, H. , … Wagner, W. (2014). Matrix elasticity, replicative senescence and DNA methylation patterns of mesenchymal stem cells. Biomaterials, 35(24), 6351–6358. 10.1016/j.biomaterials.2014.04.079 24824582

[term2843-bib-0036] Serban, A. I. , Stanca, L. , Geicu, O. I. , Munteanu, M. C. , Costache, M. , & Dinischiotu, A. (2015). Extracellular matrix is modulated in advanced glycation end products milieu via a RAGE receptor dependent pathway boosted by transforming growth factor‐beta1 RAGE. Journal of Diabetes, 7(1), 114–124. 10.1111/1753-0407.12154 24666836

[term2843-bib-0037] Sharma, P. K. , Busscher, H. J. , Terwee, T. , Koopmans, S. A. , & van Kooten, T. G. (2011). A comparative study on the viscoelastic properties of human and animal lenses. Experimental Eye Research, 93(5), 681–688. 10.1016/j.exer.2011.08.009 21910988

[term2843-bib-0038] Spiekman, M. , Przybyt, E. , Plantinga, J. A. , Gibbs, S. , van der Lei, B. , & Harmsen, M. C. (2014). Adipose tissue‐derived stromal cells inhibit TGF‐beta1‐induced differentiation of human dermal fibroblasts and keloid scar‐derived fibroblasts in a paracrine fashion. Plastic and Reconstructive Surgery, 134(4), 699–712. 10.1097/PRS.0000000000000504 25357030

[term2843-bib-0039] Spiekman, M. , van Dongen, J. A. , Willemsen, J. C. , Hoppe, D. L. , van der Lei, B. , & Harmsen, M. C. (2017). The power of fat and its adipose‐derived stromal cells: Emerging concepts for fibrotic scar treatment. Journal of Tissue Engineering and Regenerative Medicine, 11, 3220–3235. 10.1002/term.2213 28156060 PMC5724515

[term2843-bib-0040] Stasch, T. , Hoehne, J. , Huynh, T. , de Baerdemaeker, R. , Grandel, S. , & Herold, C. (2015). Debridement and autologous lipotransfer for chronic ulceration of the diabetic foot and lower limb improves wound healing. Plastic and Reconstructive Surgery, 136(6), 1357–1366. 10.1097/PRS.0000000000001819 26273734

[term2843-bib-0041] Terlizzi, V. , Kolibabka, M. , Burgess, J. K. , Hammes, H. P. , & Harmsen, M. C. (2017). The pericytic phenotype of adipose tissue‐derived stromal cells is promoted by NOTCH2. Stem Cells. 10.1002/stem.2726, 36, 240–251. 29067740

[term2843-bib-0042] Uriel, S. , Huang, J. J. , Moya, M. L. , Francis, M. E. , Wang, R. , Chang, S. Y. , … Brey, E. M. (2008). The role of adipose protein derived hydrogels in adipogenesis. Biomaterials, 29(27), 3712–3719. 10.1016/j.biomaterials.2008.05.028 18571717

[term2843-bib-0043] van Dongen, J. A. , Stevens, H. P. , Harmsen, M. C. , & van der Lei, B. (2017). Mechanical micronization of lipoaspirates: Squeeze and emulsification techniques. Plastic and Reconstructive Surgery, 139(6), 1369e–1370e. 10.1097/PRS.0000000000003372 28207564

[term2843-bib-0044] van Dongen, J. A. , Stevens, H. P. , Parvizi, M. , van der Lei, B. , & Harmsen, M. C. (2016). The fractionation of adipose tissue procedure to obtain stromal vascular fractions for regenerative purposes. Wound Repair and Regeneration, 24(6), 994–1003. 10.1111/wrr.12482 27717133

[term2843-bib-0045] van Dongen, J. A. , Tuin, A. J. , Spiekman, M. , Jansma, J. , van der Lei, B. , & Harmsen, M. C. (2017). Comparison of intraoperative procedures for isolation of clinical grade stromal vascular fraction for regenerative purposes: A systematic review. Journal of Tissue Engineering and Regenerative Medicine. 10.1002/term.2407, 12, e261–e274. 28084666

[term2843-bib-0046] Verzijl, N. , DeGroot, J. , Thorpe, S. R. , Bank, R. A. , Shaw, J. N. , Lyons, T. J. , … TeKoppele, J. M. (2000). Effect of collagen turnover on the accumulation of advanced glycation end products. The Journal of Biological Chemistry, 275(50), 39027–39031. 10.1074/jbc.M006700200 10976109

[term2843-bib-0047] Vicenti, G. , Solarino, G. , Pesce, V. , Moretti, L. , Notarnicola, A. , Carrozzo, M. , … Moretti, B. (2017). Autologous lipotransfer versus stromal vascular fraction enriched lipoinjection for diabetic foot wounds healing: A pilot study. Journal of Biological Regulators and Homeostatic Agents, 31(4 suppl 1), 141–146. 29188676

[term2843-bib-0048] Voziyan, P. , Brown, K. L. , Chetyrkin, S. , & Hudson, B. (2014). Site‐specific AGE modifications in the extracellular matrix: A role for glyoxal in protein damage in diabetes. Clinical Chemistry and Laboratory Medicine, 52(1), 39–45. 10.1515/cclm-2012-0818 23492568 PMC4104777

[term2843-bib-0049] Weidenbusch, M. , & Anders, H. J. (2012). Tissue microenvironments define and get reinforced by macrophage phenotypes in homeostasis or during inflammation, repair and fibrosis. Journal of Innate Immunity, 4(5–6), 463–477. 10.1159/000336717 22507825 PMC6741480

[term2843-bib-0050] Wetzler, C. , Kampfer, H. , Stallmeyer, B. , Pfeilschifter, J. , & Frank, S. (2000). Large and sustained induction of chemokines during impaired wound healing in the genetically diabetic mouse: Prolonged persistence of neutrophils and macrophages during the late phase of repair. The Journal of Investigative Dermatology, 115(2), 245–253. 10.1046/j.1523-1747.2000.00029.x 10951242

