# Supplementary figures and images for: Adipose tissue‐derived extracellular matrix hydrogels as a release platform for secreted paracrine factors
Source: J Tissue Eng Regen Med. 2019 Apr 15;13(6):973–85. doi: 10.1002/term.2843 (PMC6593768; doi:10.1002/term.2843)

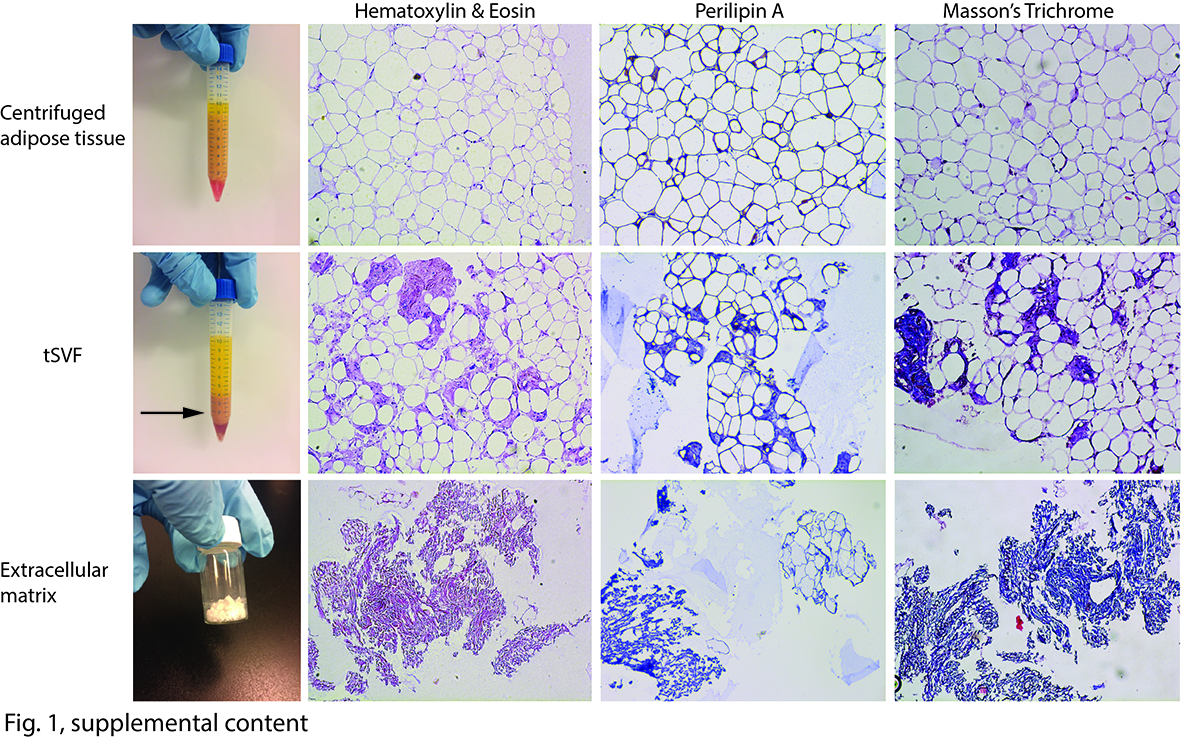

Supplement: Supplementary file 1 — Fig. S1 Light micrographs of hematoxylin & eosin staining, perilipin A staining and Masson's trichrome staining of respectively centrifuged adipose tissue, tSVF and extracellular matrix. tSVF = tissue stromal vascular fraction. [file TERM-13-973-s001.tif]

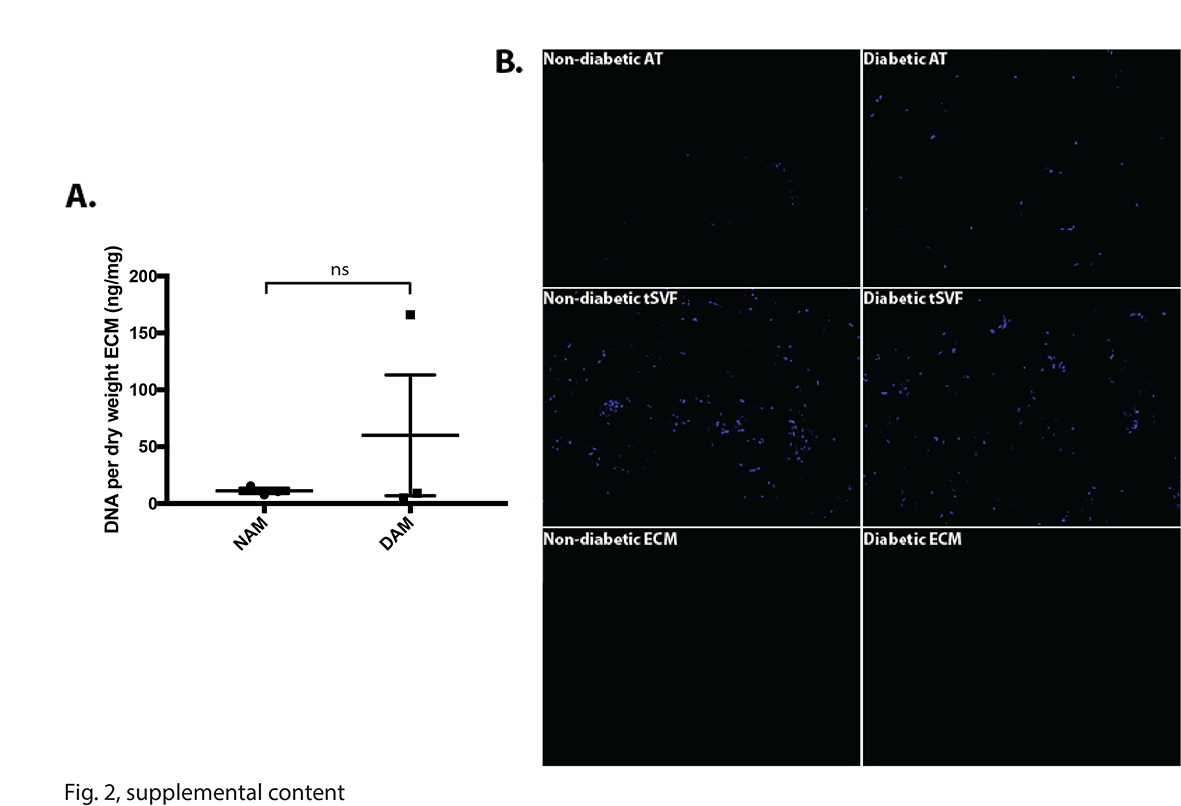

Supplement: Supplementary file 2 — Fig. S2 (A) Statistical analyses of DNA contents per dry weight ECM (ng/mg) of NAM and DAM samples (n = 3). (B) Immunofluorescent microscope photographs of DAPI staining. ECM = extracellular matrix, NAM = non‐diabetic acellular matrix, DAM = diabetic acellular matrix, AT = adipose tissue, tSVF = tissue stromal vascular fraction, NS = non‐significant. Results are expressed as mean with standard error of the mean. [file TERM-13-973-s002.tif]

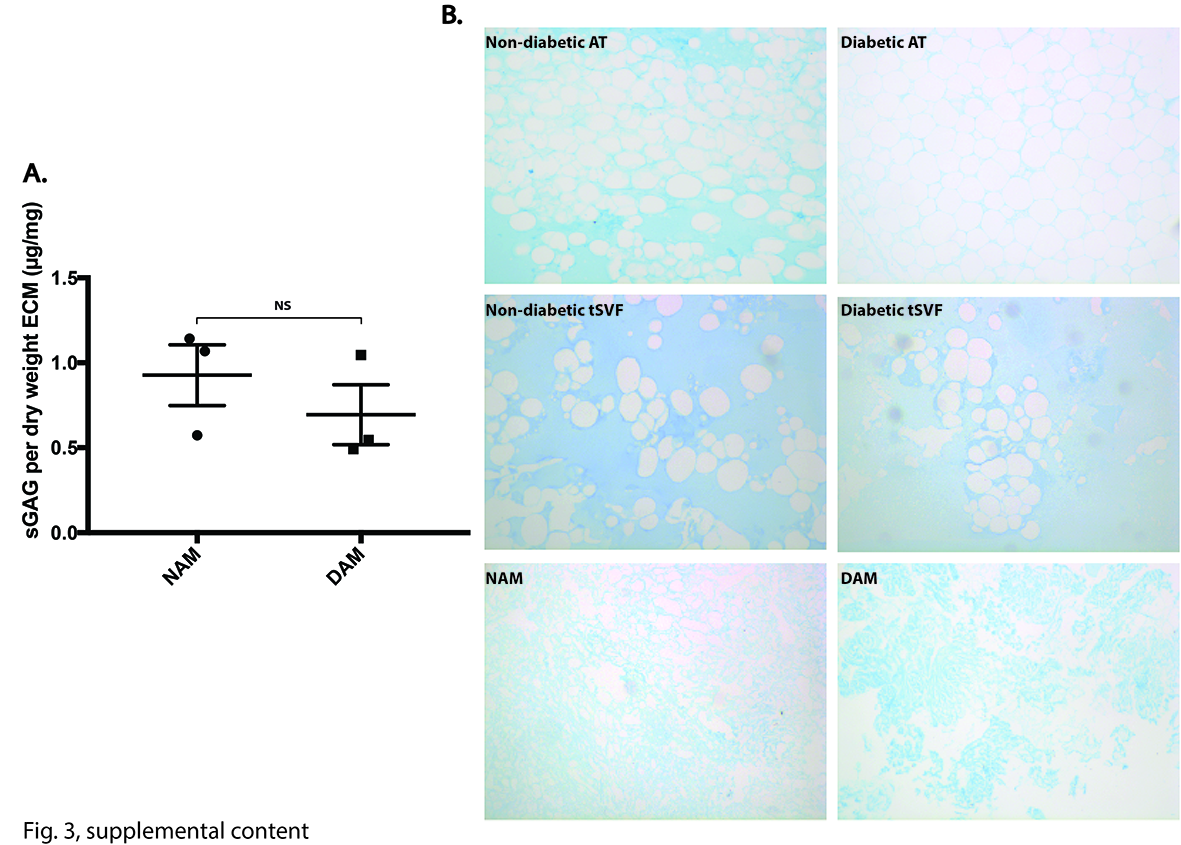

Supplement: Supplementary file 3 — Fig. S3 (A) Statistical analyses of sGAG contents per dry weight ECM (μg/mg) of NAM and DAM samples (n = 3). (B) Light microscope photographs of alcian blue staining. sGAG = sulphated glycosaminoglycan, ECM = extracellular matrix, NAM = non‐diabetic acellular matrix, DAM = diabetic acellular matrix, AT = adipose tissue, tSVF = tissue stromal vascular fraction, NS = non‐significant. Results are expressed as mean with standard error of the mean. [file TERM-13-973-s003.tif]
